# Effects of asymmetric medical insurance subsidy on hospitals competition under non-price regulation

**DOI:** 10.1186/s12939-016-0468-8

**Published:** 2016-11-15

**Authors:** Chan Wang, Pu-yan Nie

**Affiliations:** 1 Institute of Industrial Economics, Jinan University, Guangzhou, 510632 People’s Republic of China; 2 Guangdong University of Finance & Economics, Guangzhou, 510320 People’s Republic of China

**Keywords:** Quality competition, Medical insurance subsidy, Management efficiency, Game theory, I11, L51, I13

## Abstract

**Background:**

Poor medical care and high fees are two major problems in the world health care system. As a result, health care insurance system reform is a major issue in developing countries, such as China. Governments should take the effect of health care insurance system reform on the competition of hospitals into account when they practice a reform. This article aims to capture the influences of asymmetric medical insurance subsidy and the importance of medical quality to patients on hospitals competition under non-price regulation.

**Methods:**

We establish a three-stage duopoly model with quantity and quality competition. In the model, qualitative difference and asymmetric medical insurance subsidy among hospitals are considered. The government decides subsidy (or reimbursement) ratios in the first stage. Hospitals choose the quality in the second stage and then support the quantity in the third stage. We obtain our conclusions by mathematical model analyses and all the results are achieved by backward induction.

**Results:**

The importance of medical quality to patients has stronger influence on the small hospital, while subsidy has greater effect on the large hospital. Meanwhile, the importance of medical quality to patients strengthens competition, but subsidy effect weakens it. Besides, subsidy ratios difference affects the relationship between subsidy and hospital competition. Furthermore, we capture the optimal reimbursement ratio based on social welfare maximization. More importantly, this paper finds that the higher management efficiency of the medical insurance investment funds is, the higher the best subsidy ratio is.

**Conclusions:**

This paper states that subsidy is a two-edged sword. On one hand, subsidy stimulates medical demand. On the other hand, subsidy raises price and inhibits hospital competition. Therefore, government must set an appropriate subsidy ratio difference between large and small hospitals to maximize the total social welfare. For a developing country with limited medical resources and great difference in hospitals such as China, adjusting the reimbursement ratios between different level hospitals and increasing medical quality are two reasonable methods for the sustainable development of its health system.

## Background

Subsidized health insurance programs are often employed by developing countries to provide basic health care to their poor and uninsured citizens. In China, the basic medical insurance is constituted by three groups, the Medical Insurance for Urban Workers, the Urban Resident Basic Medical Insurance, and the New Rural Cooperative Medical. Those three types of insurance are supported with different levels of medical subsidies and people can only choose one of those three. Meanwhile, patients obtain diverse medical subsidies when they go to different grade hospitals. Health insurance subsidy accounts for a vast majority of expenditures of the government’s investment in the medical industry. However, poor access and high fees are still the two major problems in China’s health system.

Medical quality and medical subsidy are two important factors influencing health seeking behavior of patients. We define medical quality as cure ability and service level of the hospital and medical quantity as the number of patients a hospital treats in this study. Both medical quality and subsidy influence health-seeking behavior of patients. Medical quality has positive influence on patients because higher cure ability means the patient has higher probability to be cured and higher service level represents that patients have higher satisfaction. Medical subsidy signifies that the government will pay part of the expenditures of patients if they go to see the doctor. Many countries reimburse patients with some fixed amount. For example, for a hip replacement operation, the hospital will get 1000 USD. But under the current medical insurance policy, the Chinese government chooses to reimburse the patient with a certain ratio of his or her expenditures. Based on this medical insurance policy, the hospital will receive a proportional expenditures result in an operation or medical treatment, while the patient only needs to pay the residuary. Medical subsidy aims to reduce expenditures of the patient, so it also has positive impact on the willingness of the patient to go to a hospital when he or she is sick. Although the major purpose of medical subsidy is to reduce the costs of patients, it raises the price of health care, too. Whether the real cost of the patient is actually reduced is a question to be studied.

Based on these factors above, this study aims to capture the optimal medical subsidy policy to improve patient’s health level by employing a duopoly competition model. Two hospitals, a larger hospital and a smaller one compete in the health industry.^1^ We further assume medical quality of the larger is higher than that of the smaller one.^2^ Different subsidy policy implies different subsidy ratios in this study. We try to answer the following questions in this paper: what are the effects of medical insurance subsidy and medical quality on hospital competition? What is the optimal subsidy policy? How does the management efficiency of medical insurance funds impact the optimal subsidy ratio? The results of this study are helpful for the government of China to practice suitable health insurance policy.

## Literature review

Quality competition is a major non-price competition in industrial organization [1]. Quite a lot of prior research is focused on quality competition from many different perspectives. Ye and Mukhopadhyay [2] investigated quality competition from a demand side strategy perspective based on a duopoly model. Their study illustrated that different quality firms have different competition strategies and low quality firms prefer demand side strategy. Blair and Durrance [3] investigated the economics of collusion on quality under constraints. They showed associated effects of collusive behavior on consumer welfare. Cellini and Lamantia [4] studied the effects of minimum quality standards on quality competition under duopoly market model. Auray, Mariotti and Moizeau [5], Wang, Chen and He [6] highlighted quality regulation.

Some other studies pay much attention to hospitals because quality is quite critical in this industry. Berta et al. [7] captured the effect of imperfect quality information on hospital competition of Italy. They highlighted the effect of quality information on patients’ choice of hospitals. Somayeh, Hossein and Michael [8] also studied quality competition and they showed that patients prefer to choose high quality hospitals. Palangkaraya and Yong [9] investigated the effects of competition on hospital quality and they issued that competition either has positive or negative impacts on hospital quality, which is dependent on the measure of quality. Interestingly, Tay [10] declared that quality difference is especially important in the hospital industry and patients are not substitutes between different hospitals. Although numerous studies involve quality competition, few of them involve medical insurance reimbursement.

Besides quality competition, subsidy is widely used in many other fields [11, 12]. The impacts of subsidy under duopoly, however, are different in various sectors and fields. For instance, R&D subsidy stimulates innovation investment effectively [13, 14], but investment subsidy may crowd-out private expenditures in investment [15, 16]. Zheng Shi [17] showed that subsidy and service price may have a negative relation with Mobile telecommunication. But, Feng et al. [18] addressed that medical insurance subsidy increases medical service price. Besides, Xiong et al. [19] showed that it is an indispensable means of competition for the Internet enterprises to adopt subsidy policies. But, it will affect the growth of its profits, when subsidy policies are adopted improperly or employed without being innovated. Some researchers also studied the relationship between subsidy and quality. Sauer et al. [20] investigated the effect of environmental subsidy on quality of surface waters. In comparison, this paper captures medical insurance subsidy. But many studies focused on the effect of medical insurance subsidy on health and medical service utilization [21, 22]. However, there is little evidence on the effects of medical subsidy on quality competition.

A novel innovation of this study is in combining medical quality of hospitals and medical insurance subsidy. The major contributions of this paper are outlined as follows. First, the importance of quality to patients strengthens competition among hospitals, while medical subsidy weakens it. Second, reimbursement ratio difference between small and large hospitals affects the impact of subsidy on competition. Third, we capture the optimal reimbursement ratio based on the maximization of social welfare. Meanwhile, the higher management efficiency the medical insurance investment fund is, the higher the optimal subsidy ratios are.

The rest of this paper is organized as follows. The model under duopoly is established in the next section. Here we establish a three-stage duopoly model with quantity and quality competition. The government decides the reimbursement ratio of medical expenses in the first stage. Two hospitals choose the quality in the second stage and then support the quantity in the third stage. The model with Cournot is analyzed in Section 4. Then, some discussions are presented in the final section.

## Methods

Here, we establish a duopoly model by taking quantity and quality competition with medical reimbursement (subsidy) into account. Suppose there are two hospitals denoted as *i*∈ {*g*,*s*} in the industry.^3^ Hospitals’ medical qualities (cure ability and service level) are denoted by *q* = (*q*_*g*_,*q*_*s*_), where subscript *g* represents the large (or high quality) hospital, and *s* denotes the small (or low quality) one. Hospitals’ reimbursement ratios for their treatment and service from the government are τ = (τ_*g*_,τ_*s*_). Similarly, the quantity vector is *x* = (*x*_*g*_,*x*_*s*_) and the medical care price is *p* = (*p*_*g*_,*p*_*s*_). Then, the functions of the utility-maximizing patients as well as the profit-maximizing hospitals are introduced. In the following, we will outline the behaviors of patients and hospitals, respectively.

### Patients

The utility function of the representative patient is presented as

1 $$ U=\left(\alpha +\beta {q}_g+\lambda {\tau}_g\right){x}_g+\left(\alpha +\beta {q}_s+\lambda {\tau}_s\right){x}_s-\frac{1}{2}\left({x_g}^2+{x_s}^2\right)-\gamma {x}_g{x}_s. $$

This utility function is similar to Ferrara & Missios [23], Chen & Nie [24] and Chen & Nie [25], where the constant α > 0, parameter β > 0,λ > 0. β and λ represent the importance of quality and subsidy to patients, respectively. Quality plays a critical role in hospital industry, so we highlight the influence of the importance of quality to patients in this study. And γ ϵ [0,1] means the product substitutability. In general, the medical quality of a large hospital is higher than that of small one (*q*_*g*_ > *q*_*s*_) and subsidy ratio of the large hospital is lower than that of the small one ($\tau$_*g*_<$\tau$_*s*_). In other words, the larger hospital has a quality advantage, while the smaller one has subsidy superiority. Based on utility maximization, the corresponding inverse demand functions are:

2 $$ \begin{array}{l}{p}_g=\alpha +\beta {q}_g-{x}_g-\gamma {x}_s+\lambda {\tau}_g,\\ {}{p}_s=\alpha +\beta {q}_s-{x}_s-\gamma {x}_g+\lambda {\tau}_s.\end{array} $$

Here, we assume that prices are determined by the game between hospitals and the government. From function (2), quality and subsidy have positive influence on prices, whereas both the quantity and outputs of the rivals negatively relate to their prices. Based on functions (1) and (2), we obtain the patient surplus as follows:

3 $$ CS=U-{\displaystyle \sum_{i=g,s}\left(1-{\tau}_i\right){p}_i{x}_i}. $$

### Hospitals

Here, we model the two hospitals in this industry that offer treatment and service with different quality levels and they acquire different medical insurance funds from the government.^4^

The objective functions of the two hospitals are:

4 $$ \begin{array}{l}{\pi}_g=\left(\alpha +\beta {q}_g-{x}_g-\gamma {x}_s+\lambda {\tau}_g\right){x}_g-\frac{1}{2}\left({x_g}^2+{q_g}^2\right)-{x}_g{q}_g,\\ {}{\pi}_s=\left(\alpha +\beta {q}_s-{x}_s-\gamma {x}_g+\lambda {\tau}_s\right){x}_g-\frac{1}{2}\left({x_s}^2+{q_s}^2\right)-{x}_s{q}_s.\end{array} $$

Notice that the vector (*α* + *βq*_*g*_ − *x*_*g*_ − *γx*_*s*_ + *λτ*_*g*_)*x*_*g*_ includes medical insurance funds from the government, while $c\left({x}_i,{q}_i\right)=\frac{1}{2}\left({x_i}^2+{q_i}^2\right)-{x}_i{q}_i$ is the cost function.

The timing of the game is: In the first stage, the government declares medical reimbursement ratios. Then, the two hospitals commit to their medical quality level in the second stage. In the third stage, according to the quality levels, the hospitals decide the quantity of patient treatments and medical services, whereas patients choose the quantity of treatment and service. All solutions are obtained by backward induction, which means we will get the quantity of the last stage based on the quality and subsidy, following the quality of the second stage and then the subsidy ratio of the first stage.

Before model analysis, we make the following assumption.

**Assumption** $$ 1<\beta <\overline{\beta} $$ and $$ \underline{\gamma}<\gamma \le 1 $$.

$1<\beta <\overline{\beta}$ ensures both hospitals practice quality innovation and *q*_*g*_ > *q*_*s*_ > 0, while $\underline{\gamma}<\gamma \le 1$ indicates that the products and services of the large hospital cannot completely replace those of the small one.^5^ In other words, the two hospitals supply different treatments and services.

## Results

Here, the model under Cournot competition is analyzed. We attack the model using backward induction which means the equilibrium of the third stage is first solved. Then we address the second and the first stage.

In the third stage, by the first-order optimal conditions of function (4), we obtain

$$ \frac{\partial {\pi}_g}{\partial {x}_g}=\alpha -{q}_g+\beta {q}_g-3{x}_g-\gamma {x}_s+\lambda {\tau}_g=0. $$

5 $$ \frac{\partial {\pi}_s}{\partial {x}_s}=\alpha -{q}_s+\beta {q}_s-3{x}_s-\gamma {x}_g+\lambda {\tau}_s=0. $$

Equation (5) indicates

6 $$ \alpha -{q}_i+\beta {q}_i+\lambda {\tau}_i=3{x}_i+\gamma {x}_j. $$

Therefore, we have

7 $$ \begin{array}{l}{x}_g^{*}=\frac{-3\alpha +\alpha \gamma -3\left(-1+\beta \right){q}_g+\left(-1+\beta \right)\gamma {q}_s-3\lambda {\tau}_g+\gamma \lambda {\tau}_s}{-9+{\gamma}^2},\\ {}{x}_s^{*}=\frac{-3\alpha +\alpha \gamma -3\left(-1+\beta \right){q}_s+\left(-1+\beta \right)\gamma {q}_g-3\lambda {\tau}_s+\gamma \lambda {\tau}_g}{-9+{\gamma}^2}.\end{array} $$

For the second stage, by substituting equation (7) into function (4) and solving it, we get the equilibrium quality of the second stage.

8 $$ \begin{array}{l}{q}_g^{*}=-\frac{9\left(-1+\beta \right)\left(\alpha \left(-18-18\beta +9{\beta}^2+9\gamma +3{\gamma}^2-{\gamma}^3\right)+3\left(-6-6\beta +3{\beta}^2+{\gamma}^2\right)\lambda {\tau}_g-\gamma \left(-9+{\gamma}^2\right)\lambda {\tau}_s\right)}{324-324{\beta}^3+81{\beta}^4-189{\gamma}^2+54{\beta}^2{\gamma}^2+27{\gamma}^4-{\gamma}^6-108\beta \left(-6+{\gamma}^2\right)},\\ {}{q}_s^{*}=-\frac{9\left(-1+\beta \right)\left(\alpha \left(-18-18\beta +9{\beta}^2+9\gamma +3{\gamma}^2-{\gamma}^3\right)+3\left(-6-6\beta +3{\beta}^2+{\gamma}^2\right)\lambda {\tau}_s-\gamma \left(-9+{\gamma}^2\right)\lambda {\tau}_g\right)}{324-324{\beta}^3+81{\beta}^4-189{\gamma}^2+54{\beta}^2{\gamma}^2+27{\gamma}^4-{\gamma}^6-108\beta \left(-6+{\gamma}^2\right)}.\end{array} $$

Then we get the final equilibrium quantities by substituting equation (8) into function (7):

9 $$ \begin{array}{l}{x}_g^{*}=\frac{\left(9-{\gamma}^2\right)\left(\alpha \left(18+18\beta -9{\beta}^2-9\gamma -3{\gamma}^2+{\gamma}^3\right)-3\left(-6-6\beta +3{\beta}^2+{\gamma}^2\right)\lambda {\tau}_g+\gamma \left(-9+{\gamma}^2\right)\lambda {\tau}_s\right)}{324-324{\beta}^3+81{\beta}^4-189{\gamma}^2+54{\beta}^2{\gamma}^2+27{\gamma}^4-{\gamma}^6-108\beta \left(-6+{\gamma}^2\right)},\\ {}{x}_s^{*}=\frac{\left(9-{\gamma}^2\right)\left(\alpha \left(18+18\beta -9{\beta}^2-9\gamma -3{\gamma}^2+{\gamma}^3\right)-3\left(-6-6\beta +3{\beta}^2+{\gamma}^2\right)\lambda {\tau}_s+\gamma \left(-9+{\gamma}^2\right)\lambda {\tau}_g\right)}{324-324{\beta}^3+81{\beta}^4-189{\gamma}^2+54{\beta}^2{\gamma}^2+27{\gamma}^4-{\gamma}^6-108\beta \left(-6+{\gamma}^2\right)}.\end{array} $$

and equilibrium prices:

10 $$ \begin{array}{l}{p}_g^{*}=-\frac{\left(9+9\beta -2{\gamma}^2\right)\left(\alpha \left(-18-18\beta +9{\beta}^2+9\gamma +3{\gamma}^2-{\gamma}^3\right)+3\left(-6-6\beta +3{\beta}^2+{\gamma}^2\right)\lambda {\tau}_g-\gamma \left(-9+{\gamma}^2\right)\lambda {\tau}_s\right)}{324-324{\beta}^3+81{\beta}^4-189{\gamma}^2+54{\beta}^2{\gamma}^2+27{\gamma}^4-{\gamma}^6-108\beta \left(-6+{\gamma}^2\right)},\\ {}{p}_s^{*}=-\frac{\left(9+9\beta -2{\gamma}^2\right)\left(\alpha \left(-18-18\beta +9{\beta}^2+9\gamma +3{\gamma}^2-{\gamma}^3\right)+3\left(-6-6\beta +3{\beta}^2+{\gamma}^2\right)\lambda {\tau}_s-\gamma \left(-9+{\gamma}^2\right)\lambda {\tau}_g\right)}{324-324{\beta}^3+81{\beta}^4-189{\gamma}^2+54{\beta}^2{\gamma}^2+27{\gamma}^4-{\gamma}^6-108\beta \left(-6+{\gamma}^2\right)}.\end{array} $$

The optimal reimbursement ratios in the first stage will be provided and analyzed in the social welfare analyses section later. Next, we study effects of the reimbursement ratios on the equilibrium quantity, price and quality of hospitals.

### Medical quality analyses

Denote *Δx* = *x*_*g*_ − *x*_*s*_ the quality difference and $\frac{\varDelta x}{\varSigma x}$ the quantity deviation, here *Σx* = *x*_*g*_ + *x*_*s*_. Then from equation (9), we have the following conclusions.

**Proposition 1**: When $1<\beta <\overline{\beta}$, and $\underline{\gamma}<\gamma \le 1$, we have

$$ \begin{array}{l}(1)\ \frac{\partial {x}_s^{*}}{\partial \beta }>0,\ \frac{\partial \varSigma {x}^{*}}{\partial \beta }>0,\ \frac{\partial \varDelta {x}^{*}}{\partial \beta }<0,\ \frac{\partial \left(\frac{\varDelta {x}^{*}}{\varSigma x}\right)}{\partial \beta }<0\ \mathrm{but}\ \mathrm{sign}\left\{\frac{\partial {x}_g^{*}}{\partial \beta}\right\}\mathrm{is}\ \mathrm{ambigous};\\ {}(2)\ \frac{\partial {x}_g^{*}}{\partial \lambda }>0,\ \frac{\partial {x}_s^{*}}{\partial \lambda}\left\{\begin{array}{l}<0,\ \mathrm{if}\ 0<\frac{\tau_g}{\tau_s}<\overline{b}\\ {}>0,\ \mathrm{if}\ \overline{b}<\frac{\tau_g}{\tau_s}<1\end{array}\right.,\ \frac{\partial \varSigma {x}^{*}}{\partial \lambda }>0,\ \frac{\partial \varDelta {x}^{*}}{\partial \lambda }>0\ \mathrm{and}\ \frac{\partial \left(\frac{\varDelta {x}^{*}}{\varSigma x}\right)}{\partial \lambda }>0.\end{array} $$

**Remarks**: First, the conclusions of Proposition 1 show that the importance of medical quality to patients increases medical quantity of the small hospital, but its impact on the large hospital is uncertain. A suitable reason is that the larger hospital already gets enough patients, so the increase of the importance of medical quality to patients does not necessarily increase its patients. However, the effects of the importance of medical quality to patients on the total quantity of the two hospitals are positive. On the other hand, the effects of the importance of medical quality to patients on the quantity difference between the two hospitals are negative. It is stated that the effects of the importance of medical quality to patients on the small hospital are greater than that of the larger one. Meanwhile, we find the importance of medical quality to patients decreases quantity deviation. Those conclusions illustrate that the importance of medical quality to patients increases the symmetry of hospitals and enhances the competition as well.

Second, the subsidy effect or the importance of subsidy to patients stimulates the large hospital’s quantity, and also increases that of the small one only under the situation that the subsidy ratio difference between the two hospitals is small. Interestingly, the effect of subsidy on the quantity of the small hospital is reversed if the subsidy ratio difference is large enough, which means that the subsidy effect will fail to release effective medical needs if the subsidy ratio difference of two hospitals is too large.

In addition, we discover that the subsidy effect increases total demand. On the other hand, it expands quantity difference and quantity deviation between the large and small hospitals, too. In other words, the conclusions of Proposition 1 show that the effect of subsidy on the quantity of the large hospital is greater than that of the small one. That is why more and more patients in China prefer to choose large hospitals under the current subsidy policy [26]. Thus, the government needs to practice such policy that increases the subsidy difference between large and small hospitals to achieve a reasonable distribution of medical consumption.

### Medical price analyses

Denote by *Δp* = *p*_*g*_ − *p*_*s*_ the price difference and $\frac{\varDelta p}{\varSigma p}$ the price deviation, where *Σp* = *p*_*g*_ + *p*_*s*_. From function (10), we achieve the following conclusions.

**Proposition 2**: When $1<\beta <\overline{\beta}$ and $\underline{\gamma}<\gamma \le 1$, we have

$$ \begin{array}{l}(1)\ \frac{\partial {p}_g^{*}}{\partial \beta }>0,\ \frac{\partial {p}_s^{*}}{\partial \beta }>0,\ \frac{\partial \varSigma {p}^{*}}{\partial \beta }>0,\ \frac{\partial \varDelta {p}^{*}}{\partial \beta }<0\ \mathrm{and}\ \frac{\partial \left(\frac{\varDelta {p}^{*}}{\varSigma p}\right)}{\partial \beta }<0;\\ {}(2)\ \frac{\partial {p}_g^{*}}{\partial \lambda }>0,\ \frac{\partial {p}_s^{*}}{\partial \lambda }=\left\{\begin{array}{l}<0,\ \mathrm{if}\ 0<\frac{\tau_g}{\tau_s}<\overline{b}\\ {}>0,\ \mathrm{if}\ \overline{b}<\frac{\tau_g}{\tau_s}<1\end{array}\right.,\ \frac{\partial \varSigma {p}^{*}}{\partial \lambda }>0,\ \frac{\partial \varDelta {p}^{*}}{\partial \lambda }>0\ \mathrm{and}\ \frac{\partial \left(\frac{\varDelta {p}^{*}}{\varSigma p}\right)}{\partial \lambda }>0.\end{array} $$

**Remarks**: First, the conclusions of Proposition 2 show that the importance of medical quality to patients increases the prices of both hospitals, but reduces the price difference and price deviation. This means the importance of medical quality to patients strengthens the market power of hospitals but enhances competition, too.

Second, the subsidy effect increases the price of the larger hospital, but it increases the small hospital’s price only when the subsidy ratio difference is small. Interestingly, the subsidy effect on the price of the small hospital is reversed if the subsidy ratio difference is large enough between the two hospitals. Moreover, the effect of subsidy enlarges price difference and price deviation, which illustrates that the price increase effect of subsidy on the large hospital is larger than that on the small one. In other word, the subsidy effect increases the asymmetry between hospitals or makes hospital industry becoming more asymmetric.

Combining Proposition 1 and Proposition 2, although the subsidy effect stimulates medical purchase, it also brings the patients with some negative effects, such as making it more difficult to obtain medical service and higher medical care price.

### Medical quality analyses

Define *Δq* = *q*_*g*_ − *q*_*s*_ and $\frac{\varDelta q}{\varSigma q}$ the quality difference and deviation respectively, where *Σq* = *q*_*g*_ + *q*_*s*_. Then by equation (8), we have the following conclusions:

**Proposition 3**: When $1<\beta <\overline{\beta}$ and $\underline{\gamma}<\gamma \le 1$, we obtain

$$ \begin{array}{l}(1)\ \frac{\partial {q}_s^{*}}{\partial \beta }>0,\ \frac{\partial \varDelta {q}^{*}}{\partial \beta }<0,\ \frac{\partial \left(\frac{\varDelta {q}^{*}}{\varSigma q}\right)}{\partial \beta }<0,\ \mathrm{both}\ \mathrm{sign}\left\{\frac{\partial {q}_g^{*}}{\partial \beta}\right\}\ \mathrm{and}\ \mathrm{sign}\left\{\frac{\partial \varSigma {q}^{*}}{\partial \beta}\right\}\mathrm{are}\ \mathrm{ambigious};\\ {}(2)\ \frac{\partial {q}_g^{*}}{\partial \lambda }>0,\ \frac{\partial {q}_s^{*}}{\partial \lambda }=\left\{\begin{array}{l}<0,\ \mathrm{if}\ 0<\frac{\tau_g}{\tau_s}<\overline{b}\\ {}>0,\ \mathrm{if}\ \overline{b}<\frac{\tau_g}{\tau_s}<1\end{array}\right.,\ \frac{\partial \varSigma {q}^{*}}{\partial \lambda }>0,\ \frac{\partial \varDelta {q}^{*}}{\partial \lambda }>0\ \mathrm{and}\ \frac{\partial \left(\frac{\varDelta {q}^{*}}{\varSigma q}\right)}{\partial \lambda }>0.\end{array} $$

**Remarks**: The conclusions of Proposition 3 show that the importance of medical quality to patients increases quality innovation of the small hospital, but its effect on the quality innovation of the large hospital is ambiguous. So the effect of the importance of medical quality to patients on the total quality of the two hospitals is also ambiguous. We know that the large hospital has already owned higher quality, so the importance of medical quality to patients does not necessarily increase its quality innovation. Meanwhile, the importance of medical quality to patients decreases quality differences and quality deviation. Those conclusions indicate that increasing the importance of medical quality to patients improves quality competition between hospitals, or the conclusions imply that the enhancement of the importance of medical quality to patients will make the hospital industry more asymmetric based on quality perspective.

Furthermore, the subsidy effect stimulates quality improvement of the large hospital, and it also increases that of the small one if and only if the subsidy ratio difference is not too large. But the subsidy effect on the quality of the small hospital is negative if the subsidy ratio difference between two hospitals is large enough. Thus, the government needs to make an appropriate subsidy ratio difference between hospitals to improve medical care quality of hospitals simultaneously. In addition, we find that the subsidy effect improves the overall quality level of the industry, while it enlarges quality difference and quality deviation between large and small hospitals. It shows that the enhancement of subsidy effect improves the quality level of hospital industry, but it reduces the level of competition among hospitals at the same time. Consequently, the subsidy effect is a double-edged sword.

The conclusions from Proposition 1 to Proposition 3 indicate that subsidy ratio difference between large and small hospitals impacts the price sensitivity of the reimbursement ratio. Thus, to achieve an optimal subsidy ratio is quite critical based on the maximization of social welfare perspective.

### Social welfare analyses

In China, hospitals’ medical insurance funds are connected to the quantity of patients. The more patients a hospital has, the more medical insurance funds it receives. Therefore, whether the allocation and management efficiency of the medical insurance funds are reasonable is a valuable question to be discussed. We will try to capture the optimal subsidy ratio based on social welfare maximization.

Here, we consider social welfare. Social welfare is defined as the total surplus of the whole society members, which equal to patients surplus plus hospitals’ profits and minus reimbursement costs of medical insurance. Under Cournot competition, the social welfare is

11 $$ SW=CS+{\pi}_{\mathrm{g}}+{\pi}_s-\left(1+u\right)\left({\tau}_{\mathrm{g}}{p}_{\mathrm{g}}{x}_{\mathrm{g}}+{\tau}_{\mathrm{s}}{p}_{\mathrm{s}}{x}_{\mathrm{s}}\right). $$

In the above function, *u* represents the management efficiency of medical insurance investment funds. Higher *u* means lower management efficiency, and vice verse. Besides, (1 + *u*)(*τ*_g_ *p*_g_ *x*_g_ + *τ*_s_ *p*_s_ *x*_s_) are the total reimbursement costs of medical insurance provided by the government. We have the first-order optimal conditions as follows

12 $$ \frac{\partial SW}{\partial {\tau}_g}=0,\frac{\partial SW}{\partial {\tau}_s}=0. $$

To facilitate the calculation, we order $\gamma =\frac{2}{3},\beta =\frac{5}{2}$ below.^6^ Equation (12) has four solutions. However, only one of them has the realistic value. In other words, equation (12) has unique real solutions and they are outlined as following.

13 $$ \begin{array}{l}{\tau}_g^{*}=\frac{72075613}{579892236u}+\frac{62\alpha }{1139\lambda }-\frac{31\mathrm{B}}{579892236\sqrt{1139}{u}^2{\lambda}^2},\\ {}{\tau}_s^{*}=\frac{72075613}{579892236u}+\frac{62\alpha }{1139\lambda }+\frac{31\mathrm{B}}{579892236\sqrt{1139}{u}^2{\lambda}^2}.\end{array} $$

$$ \mathrm{Denote}\ \mathrm{B}=\sqrt{u^2{\lambda}^2\left(29053538830008u\alpha \lambda +8196753598571{\lambda}^2-343449602773200{u}^2{\alpha}^2\right)}. $$

In order to assure 0 < *τ*_g_^*^ < *τ*_s_^*^ < 1, we need $0\le \alpha <\overline{\alpha}$ and $\overline{\alpha}$ cannot be too large.^7^

We will discuss the social welfare under different statuses as follows.

If *τ* = (*τ*_g_^*^, *τ*_s_^*^) the social welfare is:

14 $$ S{W}_1=\frac{\left(5260948u\alpha -17388215\lambda \right){\left(509124u\alpha +60013\lambda \right)}^2}{531968376554691302{u}^2\lambda }. $$

If τ_*g*_ = 0,τ_*s*_ = 1, the social welfare is

15 $$ \begin{array}{l}S{W}_2=\frac{6\left(297673594{\alpha}^2+297673594\alpha \lambda +18465927480u\alpha \lambda +43355824005{\lambda}^2\right)}{1386147361}\\ {}\begin{array}{ccc}\hfill \hfill & \hfill \hfill & \hfill -\frac{6\left(489268164u{\alpha}^2+174234960900u{\lambda}^2\right)}{1386147361}\hfill \end{array}.\end{array} $$

If τ_*g*_ = 1,τ_*s*_ = 1, the social welfare is

16 $$ S{W}_3=\frac{12\left[154877{\lambda}^2-509124u{\left(\alpha +\lambda \right)}^2\right]}{1442401}. $$

If τ_*g*_ = 0,τ_*s*_ = 0, the social welfare is

17 $$ S{W}_4=\frac{1858524{\alpha}^2}{1442401}. $$

Under $0\le \alpha <\overline{\alpha}$, we have *SW*_1_ > *SW*_2_,*SW*_1_ > *SW*_3_,*SW*_1_ > *SW*_4_ and the following proposition.

**Proposition 4**: Under the condition of maximizing social welfare, there is a unique pair of optimal reimbursement ratios (*τ*_g_^*^, *τ*_s_^*^).

**Remarks**: From social welfare perspective, we capture the optimal medical reimbursement ratio, which depends on the sensitivity of price to the reimbursement ratio and management efficiency of medical insurance funds. If policymakers only consider maximizing the patient surplus, the result must be free medical care. However, as a developing country with a great population, the government has a limited ability to bear such huge medical expenditures. But social welfare maximization is a suitable target for the policymaker when he tries to carry out a reasonable medical subsidy policy.

### Management efficiency analyses

Here, we will consider the effects of the management efficiency of medical insurance funds on optimal reimbursement ratios. The smaller *u* is, the higher the management efficiency of the medical insurance funds is. From equation (13), we get the following proposition.

Proposition 5: $$ \frac{\partial {\tau}_g^{*}}{\partial u}<0,\frac{\partial {\tau}_s^{*}}{\partial u}<0. $$

**Remarks**: The conclusions of Proposition 5 imply that improvement of the management efficiency of the medical insurance funds increases optimal medical reimbursement ratios of both hospitals, which means efficiency increase enables the government to raise the subsidy density. If the government does not focus on the efficiency of the medical insurance funds, patients cannot maximize their use of the government’s input. The conclusions of Proposition 5 are very interesting because they show that management efficiency improvement has triple advantages: (a) lowering the costs of insurance management, (b) decreasing the expenditures of patients and (c) raising the social welfare. That is why the government should sufficiently focus its attention on the management efficiency of medical insurance funds.

## Discussions

Considering the importance of quality and subsidy to patients in the hospital industry, this paper analyzes both quality and quantity competition. By employing a three-stage dynamic game model under Cournot competition, we argue that the importance of quality to patients has stronger influence on the small hospital than the larger one, but on the contrary, subsidy has greater effect on the large hospital than the small one. Furthermore, the importance of quality to patients strengthens competition among hospitals, while subsidy weakens competition. This paper also shows that reimbursement ratios difference between the small and the large hospital impacts the relationship between subsidy and competition. Thus we capture the optimal reimbursement ratio based on social welfare maximization. Meanwhile, the higher management efficiency of the medical insurance investment funds is, the higher the optimal subsidy ratios are.

Under the current subsidy policy, medical quality of larger hospitals is higher than small ones, but subsidy ratios have little difference. In other words, patients obtain higher medical quality but little costs difference by choosing larger hospitals. That is why more and more patients choose large hospitals as Wang and Chen [26] issued and that lead to great treatment pressure for larger hospitals. So it is necessary for the government to adjust subsidy difference to redistribute patients between large and small hospitals. By doing that, overload treatment pressure of larger hospitals can be relieved.

Of course, there are also some shortcomings of this study. First, this paper assumes that the influences of the importance of quality to patients and subsidy effects for different hospitals are the same. But in reality, different size hospitals may have different price increasing abilities because of difference in reputation. Second, this study only considers Cournot competition, while other competition structures such as Stacklberg competition are also common in fact. Furthermore, we focus our attention on ratio subsidy, different form China, many countries practice fixed amount reimbursement policy. So it is valuable to take those factors into account in further research and some other interesting conclusions will be achieved. Besides, nearly all hospitals have capacity constraints (limited beds and nurses) and it is important to reveal what will happen if capacity constraints are taken into consideration.

## Conclusions

Poor medical care level and high fees are the two major problems in the world health system. This paper states that subsidy is a two-edged sword. On one hand, subsidy stimulates medical demands. On the other hand, subsidy raises price and inhibits hospital competition. Therefore, the government must carry out appropriate subsidy policy and a suitable ratios difference between large and small hospitals to maximize the total social welfare.

More importantly, the government should improve its funds management efficiency. Health insurance funds are managed by different departments and have high operating costs in China. If the management efficiency of the medical insurance funds is improved, the advantage of reimbursement for patients could be further enhanced.

In China, almost all residents are covered by the ongoing basic medical insurance system and the basic medical insurance system is included by the Medical Insurance for Urban Workers, the Urban Resident Basic Medical Insurance and the New Rural Cooperative Medical. However, different groups are treated with different subsidy. As part of the ongoing health insurance system reform, the Chinese central government plans to unify the three different types of medical insurances under a unitize standard and to enlarge the reimbursement difference between different grade of hospitals. Our study shows that as developing country with limited medical resources and great differences in hospitals, it is reasonable for the Chinese government to adjust the reimbursement ratios between different hospitals and to increase medical quality as two reasonable methods for a sustainable development of its health system and a batter distribution of medical resources.

## Footnotes

1 Larger hospital and small hospital can also be treated as larger hospitals group and small hospitals group. So conclusions of this study are easy to expand to other market structure.

2 This is a reasonable assumption because in China, most of hospitals are state owned, quite a few are private hospitals and larger hospitals also mean higher medical quality.

3 Although we employ a duopoly model in this study, the conclusions are easy to extend to multi-competition situation.

4 Those medical insurance funds are actually reimbursements or subsidies from the government to patients.

5 Based on the cost function $c\left({x}_i,{q}_i\right)=\frac{1}{2}\left({x_i}^2+{q_i}^2\right)-{x}_i{q}_i$, we know that β should be larger than 1, otherwise *βx*_*i*_ *q*_*i*_ − *x*_*i*_ *q*_*i*_ < 0 and no hospital is willing to offer a positive quality. $\beta <\overline{\beta}$ indicates the importance of quality to patients or price increase ability of the quality should not be too strong, or the disadvantaged hospital will be forced to quit the competition.

6 Too many parameters lead it is impossible to make any analyses about the optimal ratios. We only care about the management efficiency of medical insurance, we β and γ are given by certain value.

7 Different from the standard linear inverse demand function *p*_*i*_ = *α* − *x*_*i*_ − *γx*_*j*_ of Cournot, price function in this study is *p*_*i*_ = *α* + *βq*_*i*_ + *λτ*_*i*_ − *x*_*i*_ − *γx*_*j*_, so α should not too large, or the price will be too high.
